# Deciphering resistance to *Zymoseptoria tritici* in the Tunisian durum wheat landrace accession ‘Agili39’

**DOI:** 10.1186/s12864-022-08560-2

**Published:** 2022-05-17

**Authors:** Sahbi Ferjaoui, Lamia Aouini, Rim B. Slimane, Karim Ammar, Suzanne Dreisigacker, Henk J. Schouten, Suraj Sapkota, Bochra A. Bahri, Sarrah Ben M’Barek, Richard G. F. Visser, Gert H. J. Kema, Sonia Hamza

**Affiliations:** 1 Laboratory of Bioaggressors and Integrated Protection in Agriculture (BPIA), National Institute of Agronomy of Tunisia (INAT), 43 Avenue Charles Nicolle, 1082 El Mahrajène, Tunis, Tunisia; 2 Present Address Field Crops Laboratory, Regional Field Crops Research Center of Beja (CRRGC), P.O. Box 9000, Beja, Tunisia; 3 Bio-Interaction and Plant Health, Wageningen University and Research, PO Box 16, 6700AA Wageningen, The Netherlands; 4 The Graduate School ‘Experimental Plant Sciences’ (EPS), Wageningen Campus, 6708 PB Wageningen, The Netherlands; 5 Present Address Center for Desert Agriculture, Biological and Environmental Science and Engineering Division, King Abdullah University of Science and Technology, Thuwal, Saudi Arabia; 6 Present address Higher Institute of Agronomy of Chott Meriam (ISA-CM), 4042 Sousse, Tunisia; 7 International Maize and Wheat Improvement Center (CIMMYT), Apdo. Postal 6‑641, 06600 Mexico, D.F. Mexico; 8 Plant Breeding, Wageningen University and Research, P.O. Box 386, 6700 AJ Wageningen, The Netherlands; 9 Institute of Plant Breeding, Genetics and Genomics, Department of Plant Pathology and Institute of Plant Breeding, University of Georgia, Griffin, GA 30223 USA; 10 Present Address United States Department of Agriculture USDA, Crop Genetics and Breeding Research Unit, Tifton, GA USA; 11 CRP-Wheat Septoria Phenotyping Platform (CIMMYT-IRESA), Regional Field Crops Research Center of Beja (CRRGC), BP 350, 9000 Beja, Tunisia; 12 Laboratory of Phytopathology, Wageningen University and Research, PO box 16, 6700AA Wageningen, The Netherlands

**Keywords:** Durum wheat, Landraces, *Zymoseptoria tritici*, *Mycosphaerella graminicola* quantitative trait loci (QTL), Gene efficacy, QTL epistasis, Gene pyramiding

## Abstract

**Background:**

Septoria tritici blotch (STB), caused by *Zymoseptoria tritici* (*Z. tritici*)*,* is an important biotic threat to durum wheat in the entire Mediterranean Basin. Although most durum wheat cultivars are susceptible to *Z. tritici*, research in STB resistance in durum wheat has been limited.

**Results:**

In our study, we have identified resistance to a wide array of *Z. tritici* isolates in the Tunisian durum wheat landrace accession ‘Agili39’. Subsequently, a recombinant inbred population was developed and tested under greenhouse conditions at the seedling stage with eight *Z. tritici* isolates and for five years under field conditions with three *Z. tritici* isolates. Mapping of quantitative trait loci (QTL) resulted in the identification of two major QTL on chromosome 2B designated as *Qstb2B_1* and *Qstb2B_2*. The *Qstb2B_1* QTL was mapped at the seedling and the adult plant stage (highest LOD 33.9, explained variance 61.6%), conferring an effective resistance against five *Z. tritici* isolates. The *Qstb2B_2* conferred adult plant resistance (highest LOD 32.9, explained variance 42%) and has been effective at the field trials against two *Z. tritici* isolates. The physical positions of the flanking markers linked to *Qstb2B_1* and *Qstb2B_2* indicate that these two QTL are 5 Mb apart. In addition, we identified two minor QTL on chromosomes 1A (*Qstb1A*) and chromosome 7A (*Qstb7A*) (highest LODs 4.6 and 4.0, and explained variances of 16% and 9%, respectively) that were specific to three and one *Z. tritici* isolates, respectively. All identified QTL were derived from the landrace accession Agili39 that represents a valuable source for STB resistance in durum wheat.

**Conclusion:**

This study demonstrates that *Z. tritici* resistance in the ‘Agili39’ landrace accession is controlled by two minor and two major QTL acting in an additive mode. We also provide evidence that the broad efficacy of the resistance to STB in ‘Agili 39’ is due to a natural pyramiding of these QTL. A sustainable use of this *Z. tritici* resistance source and a positive selection of the linked markers to the identified QTL will greatly support effective breeding for *Z. tritici* resistance in durum wheat.

**Supplementary Information:**

The online version contains supplementary material available at 10.1186/s12864-022-08560-2.

## Background

Wheat has been, for centuries, the prime food and feed crop especially in the Mediterranean basin [1]. This staple crop supplies 20% of the human calorie intake, and is thereby a major component for global food security [2, 3]. The genus *Triticum* L. comprises several wheat species with various ploidy levels, but global wheat production is almost entirely based on bread wheat, *T. aestivum* L. em. Thell. (2n = 6x = 42, sub-genomes AABBDD), and durum wheat, *T*. *turgidum L.* var. *durum* (2n = 4x = 28, sub-genomes AABB), also known as pasta wheat [4]. Durum wheat accounts for about 8% to the global wheat production, and its cultivation is concentrated in latitudes ranging from 55°N to 40°S [5, 6], corresponding mostly to the Mediterranean Basin, the North American Great Plains, India and the former USSR [6]. Durum wheat is also produced in sub-Saharan Africa (SSA) where Ethiopia is a leading country and considered as one of the biggest durum wheat producers with approximately 0.6 million ha [7], and a center of diversity for tetraploid wheat [8]. Northern Africa has been also the cradle of wheat production for centuries and was the breadbasket for the Roman Empire [9, 10] with locations such as Dougga in Tunisia, as exquisite trading zones for wheat and other commodities until the late 500’s AD [11]. In Tunisia, durum wheat occupies 725 Mha approximates representing 49% of the total annual cereal area [12], with an average yield estimated at 1.7 tons per hectare between the cropping seasons 2014/2015 and 2019/2020 [13].

Alike bread wheat, durum wheat production is significantly affected by abiotic stress conditions—mostly drought—and by the emergence of more aggressive pathogens [14]. Throughout Maghreb region, the foliar blight septoria tritici blotch (STB), caused by the hemibiotroph *Zymoseptoria tritici* (Desm.) Quaedvlieg & Crous (formerly *Mycosphaerella graminicola* (Fuckel) J. Schröt. in Cohn), is among the major threats [15]. Estimated yield losses amounted up to 385 kg.ha^−1^ in 2008–2009, which is more than 30% in most regions [16]. Recent research increased the general understanding of the *Z. tritici* epidemiology in the Maghreb. Hamada [17] reported the occurrence of the teleomorph of the fungus in Tunisia, despite the arid conditions in the region, and Neddaf et al. [18] determined an equal distribution of both mating types in Algeria, indicating regular sexual reproduction, which likely contributes to the vast genetic diversity in this region. Similar results have recently been reported in Tunisia on durum wheat [19]. The use of fungicides has been more slowly adopted by durum wheat growers as compared to bread wheat producers in Europe, and the first occurrence of strobilurin resistance have been reported in Tunisia and Algeria [18, 20].

One of the best management strategies for all plant diseases is the generation of new disease resistant germplasm through plant breeding. The huge genetic diversity in wheat and its ancestors has provided new varieties for almost a century [4]. Releasing new resistant germplasm has proven its efficacy and has turned the potential havoc of re-emerging and upcoming threats into a manageable problem [21, 22], such as the stem rust caused by the Ug99 strain [23–25]. Before modern plant breeding, improved crops frequently resulted from farmers’ selections of outperforming genotypes in terms of yield stability. Often, such so-called landraces contained a variety of closely related lines that quenched biotic threats. During the onset of breeding, these landraces were often the starting material for targeted efforts to improve for instance disease resistance [26–28].

Several studies have revealed that durum wheat landraces are a valuable source of resistance alleles against fungal pathogens [8, 12, 29–31]. STB resistance sources on durum wheat were identified in many countries such as Tunisia [12], Ethiopia [8], Iran [32] and Spain [33]. Until now, up to 22 septoria tritici blotch (*Stb*) resistance genes have been identified and mapped [34, 35]. However, due to the apparent dichotomy in natural *Z. tritici* populations for either bread wheat or durum wheat [36–38], the presence of these mapped *Stb* genes in durum wheat cannot be determined using well characterized *Z. tritici* strains originating from bread wheat. Thus far, the substantial research progress is mainly based on the *Z. tritici* – bread wheat pathosystem [34, 39]. Therefore, and albeit the growing efforts to dissect the durum wheat – *Z. tritici* interactions [12, 33, 40, 41], resistance breeding to *Z. tritici* in durum wheat has slowly progressed over the last 25 years compared to bread wheat [34]. This affects many small growers who produce this wheat as a staple crop in an area that is severely struck by septoria tritici blotch.

In this study, we have embarked on increasing the understanding of the *Z. tritici*—durum wheat pathosystem. Here, we have first screened eight durum wheat Tunisian landrace accessions for *Z. tritici* resistance. Subsequently, we developed a mapping population between the resistant landrace accession ‘Agili39’ and the susceptible modern cv. Khiar to identify the genetic basis of resistance to *Z. tritici* and to map the underlying genes under greenhouse and field conditions.

## Results

### Phenotyping of RILs, landrace accessions and modern cultivars at the seedling stage

The 20 *Z. tritici* isolates grew successfully under laboratory conditions enabling appropriate inoculum production and phenotyping. None of the tested durum landraces and cultivars was resistant to the entire suite of *Z. tritici* isolates (Table 1), but the landrace accessions showed a broader efficacy compared to the cvs. Khiar and Karim, resulting in a significant ‘line x isolate’ interaction, indicating specific gene action (Additional Table 1). Interestingly, necrosis values were high and ranged between 72 and 97% (data not shown). The parents of the developed RILs, ‘Agili39’ and cv. Khiar showed highly significantly different pycnidia values of 6% and 36%, respectively (Table 1), and henceforward, we selected a set of eight *Z. tritici* isolates that discriminated between ‘Agili39’ and cv. Khiar for subsequent phenotyping of the developed F6 RILs population (Table 2).

**Table 1:**
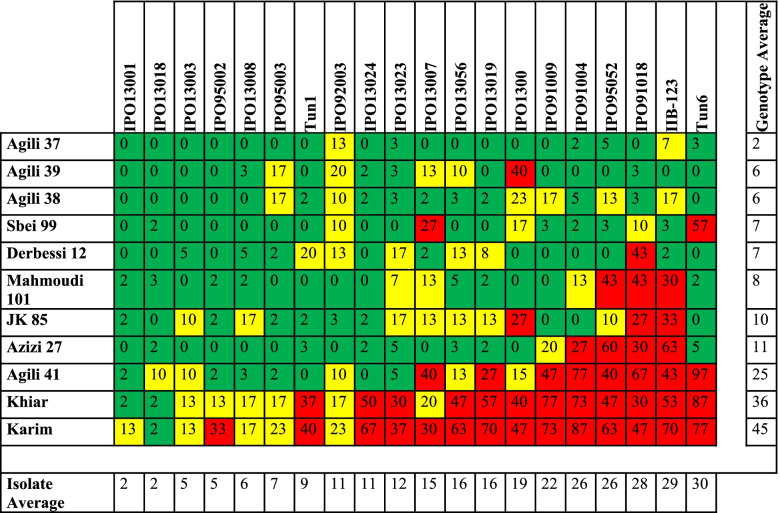
Percentage of pycnidia on the sprayed-inoculated primary leaves of durum wheat landraces and cultivars with 20 *Zymoseptoria tritici* isolates at 21 days-post inoculations. Coloured cells indicate least significant differences (LSDs; *P* = 0.05) with resistant in green (not significantly different from 0% Pycnidia), intermediate in yellow (significantly different from 0% Pycnidia and 100% Pycnidia) and susceptible in red (not significantly different from 100% Pycnidia)

**Table 2:** Origin of 20 *Zymoseptoria tritici* isolates that were isolated from durum wheat in the Mediterranean Basin and that were used for phenotyping in the seedling and adult plant stage

					Experiment		
Region	Isolate ID	Country	Location	Year	1	2	3
Middle-East	IPO91004	Syria	Lattakia	1991	+	+	
	IPO95002	Syria	Lattakia	1995	+		
	IPO95003	Syria	Lattakia	1995	+		
North Africa	IPO91009	Tunisia	Bejá	1991	+	+	
	IIB-123	Tunisia	Bejá	2005	+	+	+
	Tun1	Tunisia	Qued bagrat	-	+	+	+
	Tun6	Tunisia	Sidi Nsir	-	+	+	+
	IPO91018	Morocco	Jenica Shaim	1991	+	+	
	IPO95052	Algeria	Berrahal	1995	+	+	
Europe	IPO92003	Portugal	-	1992	+	+	
	IPO13001	Italy	Emilia Romagna	2013	+		
	IPO13003	Italy	Emilia Romagna	2013	+		
	IPO13006	Italy	Emilia Romagna	2013	+		
	IPO13007	Italy	Emilia Romagna	2013	+		
	IPO13008	Italy	Emilia Romagna	2013	+		
	IPO13018	Italy	Sicily	2013	+		
	IPO13019	Italy	Sicily	2013	+		
	IPO13023	Italy	Sicily	2013	+		
	IPO13024	Italy	Sicily	2013	+		
	IPO13056	Italy	Tuscany	2013	+		

Experiment 1 = Pre-screening of the “Agili39” landrace and the cv. Khiar with 20 *Zymoseptoria tritici* isolates under controlled conditions

Experiment 2 = Screening of the F6 “Agili39”/Khiar recombinant inbred lines with 8 *Zymoseptoria tritici* isolates under controlled conditions

Experiment 3 = Screening of the F6 “Agili39”/Khiar recombinant Inbred population with 3 *Zymoseptoria tritici* isolates under field conditions

**Fig. 1:**
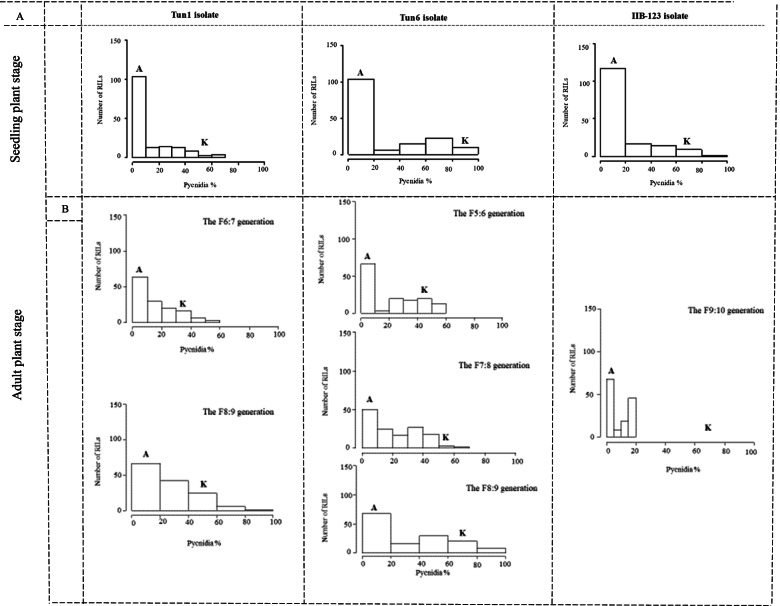
Frequency distributions of the disease severity assessed as pycnidia percentage in seedlings and adult plants of the F6-F10 recombinant inbred lines of Agili39/Khiar with three Zymoseptoria tritici under field and controlled conditions. ‘A’ and ‘K’ are referring to ‘Agili39’ and cv. Khiar parents, respectively

The seedling screening of the RILs with the selected eight *Z. tritici* isolates resulted in non-symmetric frequency distributions skewed towards the resistance phenotype for all tested isolates (Fig. 1 panel A, Additional Fig. 1). Subsequent analyses of variance of the split-plot design seedling experiment revealed that the ‘RIL’ term was highly significant for necrosis and pycnidia AUDPC scores at *p* = 0.0001 (Table 3). This result indicates that the observed variation in the data is accounted for the variable genetic make-up of the tested lines.

**Table 3:** Analysis of variance of necrosis and pycnidia AUDPC (N-AUDPC and P-AUDPC) seedling data from the ‘Agili39’/Khiar recombinant inbred lines (RILs) tested following a split-plot design

Source of variation	df^a^	Mean of Square		F value		Pr(> F)	
		**N-AUDPC**	**P-AUDPC**	**N-AUDPC**	**P-AUDPC**	**N-AUDPC**	**P-AUDPC**
**Replicate**	2	5,303,251	834,974	2.2345	2.8532	0.14966	0.09689
**Isolate**	7	2,092,146	167,005	0.8815	0.5707	0.54841	0.7668
**Error a (Main plot)**	12	2,373,376	292,643				
**RIL**	160	217,971	125,635	12.3639	13.2979	< 2.00E-16***	< 2.00E-16***
**Isolate x RIL**	1099	16,572	8909	0.94	0.943	0.87956	0.86706
**Error b (Sub-plot)**	2183	17,630	9448				

Signifiance codes: 0 ‘***’ 0.001 ‘**’ 0.01 ‘*’ 0.05 ‘.’ 0.1 ‘’ 1

^a^Degrees of freedom

Differentiation between the isolates was observed for necrosis and pycnidia scores in the RIL population tested at the seedling stage (Table 4). The highest population mean necrosis coverage (60.60%) was registered for RILs inoculated with IPO92003 isolate (Table 4). However, the least population mean necrosis coverage (34.5%) was observed for RILs inoculated with Tun1 isolate. Pycnidia population mean scores were also variable and were high for lines inoculated with Tun6 isolate (23.9%), but relatively low for lines inoculated with IPO95052 isolate (9.4%). For all tested isolates, necrosis scores ranged between 0 and 100%, however a maximum of 90% of pycnidia score was registered for lines inoculated with IPO95052 isolate (Table 4). All seedling phenotypic traits were repeatable with the highest repeatability registered for isolates IPO92003 and Tun6 for necrosis and pycnidia scores, respectively and with an equal repeatability of 0.96 for both seedling phenotypic traits and isolates (Table 4).

**Table 4:** Mean, range (minimum and maximum) and repeatability / heritability (*H*^*2*^) of Necrosis and Pycnidia values of the durum parents ‘Agili39’ and cv. Khiar and their derived recombinant inbred lines at the seedling and the adult plant stages

Isolate	Parent	Mean (± Std^a^)		RILs Physiological stage	Population mean (± Std^a^)		Range (Min–Max)		*Repeatability / H*^*2b*^	
	Trait	Necrosis %	Pycnidia %	Trait	Necrosis %	Pycnidia %	Necrosis %	Pycnidia %	Necrosis	Pycnidia
Tun1	Agili39	0.0 ± 0.5	0.0 ± 0.8	Adult-F7		15.40 ± 1.7		0.00- 51.60		0.88 ^b^
	Khiar	70.0 ± 5.0	46.7 ± 3.5	Adult-F9		21.50 ± 2.2		0.00—82.40		
				Seedling	34.50 ± 2.2	12.80 ± 3.2	0.00—90.00	0.00—70.00	0.85	0.82
Tun6	Agili39	6.7 ± 4.2	0.0 ± 1.8	Adult-F6		19.60 ± 2.1		0.0—55.3		0.98 ^b^
				Adult-F8		19.00 ± 1.9		0.00- 60.30		
	Khiar	90.0 ± 0.5	0.0 ± 2.5	Adult-F9		29.90 ± 3.8		0.00—87.30		
				Seedling	35.30 ± 3.0	23.90 ± 3.2	0.00—100.00	0.00—93.30	0.93	0.96
IIB123	Agili39	1.7 ± 0.2	0.0 ± 3.5	Adullt-F10		6.50 ± 0.94		0.00—19.40		0.97
	Khiar	90.0 ± 5.0	70.0 ± 2.6	Seedling	42.10 ± 2.9	14.00 ± 2.2	0.00—100.00	0.00 -100.00	0.92	0.91
IPO91004	Agili39	23.3 ± 0.6	0.0 ± 2.3	Seedling	40.50 ± 2.7	17.00 ± 2.5	0.00—90.00	0.00—86.70	0.91	0.93
	Khiar	80.0 ± 1.5	80.0 ± 5.3							
IPO91009	Agili39	1.7 ± 0.5	0.0 ± 3.0	Seedling	30.50 ± 2.4	9.70 ± 1.9	0.00—93.30	0.00—80.00	0.88	0.85
	Khiar	66.7 ± 3.5	56.7 ± 1.5							
IPO91018	Agili39	11.7 ± 5.3	0.0 ± 0.2	Seedling	41.80 ± 2.2	10.80 ± 1.8	0.00—90.00	0.00—76.70	0.92	0.89
	Khiar	80.0 ± 3.2	70.0 ± 3.5							
IPO92003	Agili39	66.7 ± 5.3	13.3 ± 0.2	Seedling	60.60 ± 1.5	18.40 ± 1.1	15.00—100.00	0.00—63.30	0.96	0.87
	Khiar	50.0 ± 3.4	5.0 ± 1.8							
IPO95052	Agili39	5.3 ± 2.6	0.0 ± 1.5	Seedling	36.40 ± 3.1	9.40 ± 2.1	0.00—100.00	0.00—90.00	0.92	0.89
	Khiar	70.0 ± 1.6	80.0 ± 2.5							

^a^Standard deviation

^b^Broad sense heritability

### Phenotyping of RILs at the adult plant stage

During all field trials, STB developed well after the inoculations, but only pycnidia coverage was assessed. The susceptible parent cv. Khiar showed high disease severities throughout the trials, whereas ‘Agili39’ remained free of disease (0 pycnidia) (Fig. 1 panel B). The three-way analysis of variance revealed that the ‘genotype’, ‘isolate’ and their interaction ‘genotype x isolate’ terms are highly significant at *p* = 0.0001 (Table 5). This result indicates a ‘genotype x isolate’ specificity at the adult plant. The term ‘year’ was significant at *p* = 0.05. However, the term ‘block’, the two-way interaction terms ‘genotype x block’ and ‘genotype x year’, and the three-way interaction term ‘genotype x year x isolate’ were not significant, indicating no variation in the micro-environment and the homogeneity of the field inoculation (Table 5).

**Table 5:** Analysis of variance for adult plant disease severity scores in the F6-F9 ‘Agili39’/Khiar recombinant inbred lines (RILs) that were inoculated with *Zymoseptoria tritici* isolates Tun1 and Tun6

Source of Variance	Df^a^	Mean of Square	F value	Pr(> F)
Genotype	174	4283	9.439	< 2e-16 ***
Isolate	1	20,847	45.94	2.18e-09 ***
Year	3	1721	3.793	0.013581 *
Block	4	300	0.661	0.620963
Genotype x Block	51	262	0.578	0.980775
Genotype x Isolate	167	955	2.105	0.000165 ***
Genotype x Year	465	162	0.356	1
Genotype x Year x Isolate	653	365	0.89	0.80147
Residuals	77	454		

Signifiance codes: 0 ‘***’ 0.001 ‘**’ 0.01 ‘*’ 0.05 ‘.’ 0.1 ‘’ 1

^a^Degrees of freedom

Overall, adult plants F9 showed the highest average of pycnidia coverage compared to the F7 inoculated with the Tun1 isolate (21.5% of pycnidia for the F9 compared to 15.4% for the F7), and to the F6 and the F8 generations inoculated with the Tun6 isolate (29.9% of pycnidia for the F9 compared to 19.6% and 19.0% for the F6 and the F8, respectively) (Table 4, Fig. 1 panel B). The field disease severity on the F9 generation inoculated with Tun1 and Tun6 isolates was also variable with a higher severity for Tun6 isolate (29.9%) compared to the Tun1 isolate (21.5%), which indicates once more a specific ‘genotype x isolate’ interaction. Interestingly, average pycnidia coverage was relatively low at the F10 generation inoculated with the IIB-123 isolate (6.5% of pycnidia), with a maximum pycnidia coverage of 19.4% (Table 4).

Adult pycnidia coverage heritability (*H*^*2*^) was high for the Tun1 and the Tun6 isolates, with a higher heritability for the adult pycnidia coverage caused by the Tun6 isolate (*H*^*2*^ = 0.98) compared to the heritability of the pycnidia coverage caused by the Tun1 isolate (*H*^*2*^ = 0.88) (Table 4). Field data generated by the inoculation of the F10 RIL with the IIB123 isolate were also repeatable (0.97) (Table 4).

### Correlations between the seedling and the adult plant assays

**Fig. 2:**
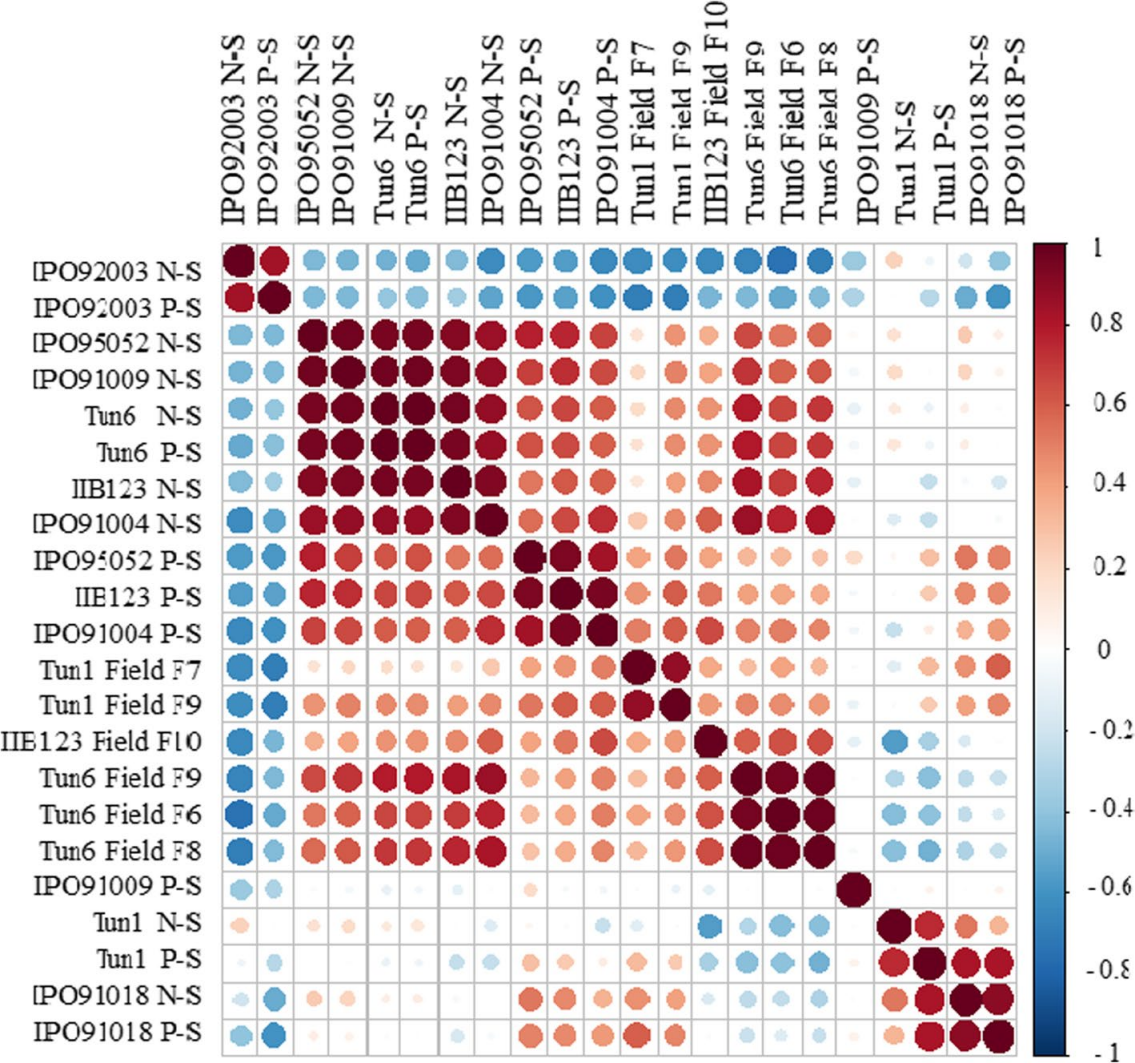
Heatmap correlation between the *Zymoseptoria tritici* isolates tested under field and controlled conditions on the ‘Agili39’/Khiar mapping population performed using the “Corr” function and visualized using the “corrplot” package in the R environment [42, 43]. Different traits are named by the isolate followed by the Necrosis (N) or pycnidia (P) development at the seedling stage (S). Isolates tested at the adult plant stage are followed by the term field, and the RILs advanced generation (F6 – F10). Colours gradient is an indication of high (red colour) and low (blue colour) correlation between traits

Low to high correlations were observed between the different traits (Fig. 2, Additional Table 2). Phenotypic scores obtained by the IPO92003 isolate were the least correlated with all phenotypic scores obtained by the tested isolates at the seedling and the adult plant stages (Fig. 2, Additional Table 2). Nonetheless, necrosis and pycnidia AUDPC scores generated by the IPO92003 isolate on the tested RILs at the seedling stage were highly correlated (*r* = 0.6). *Z. tritici* isolates IPO95052, IPO91009, Tun6, IIB-123 and IPO91004 tested at the seedling stage were correlated between each other for necrosis and pycnidia AUDPC scores (0.3 < *r* > 0.5) (Fig. 2, Additional Table 2). The highest correlation coefficient (*r*) of 0.9 was registered between pycnidia AUDPC scores generated on lines inoculated by the IPO91004 and the II-B123 *Z. tritici* isolates at the seedling stage. In contrast, necrosis and pycnidia scores were rather moderately correlated between IPO91018, IIB-123, IPO95052 and IP91004 isolates with a maximum *r* of 0.5 (Additional Table 2). Field data generated by Tun6 isolate were highly correlated across the F6 – F9 generation (*r* = 0.8) (Additional Table 2). Interestingly, positive correlations were also observed between adult and seedling disease scores induced by Tun6 isolate (Additional Table 2). Similarly, positive correlations were registered between field scores generated by Tun6, and II-B123 isolates, with a correlation coefficient *r* = 0.4 (Fig. 2, Additional Table 2).

### The ‘Agili39’/ Khiar linkage genetic map and the identification of QTL for Z. tritici resistance

The ‘Agili39’/Khiar genetic linkage map consisted of 1959 *SNP* markers assigned to 30 linkage groups representative of the 14 durum wheat chromosomes (Additional Table 3). The total length of the genetic map was 4220 cM. In average, linkage groups consisted of 80.83 *SNP* markers spanning an average length of 140.7 cM, in which adjacent loci were in average 2.3 cM distant (Additional Table 3). Linkage group sizes ranged from 28.7 cM and 338.0 cM, corresponding to linkage groups 12 and 7, respectively, both representative of chromosome 1B (Additional Table 3).

**Fig. 3:**
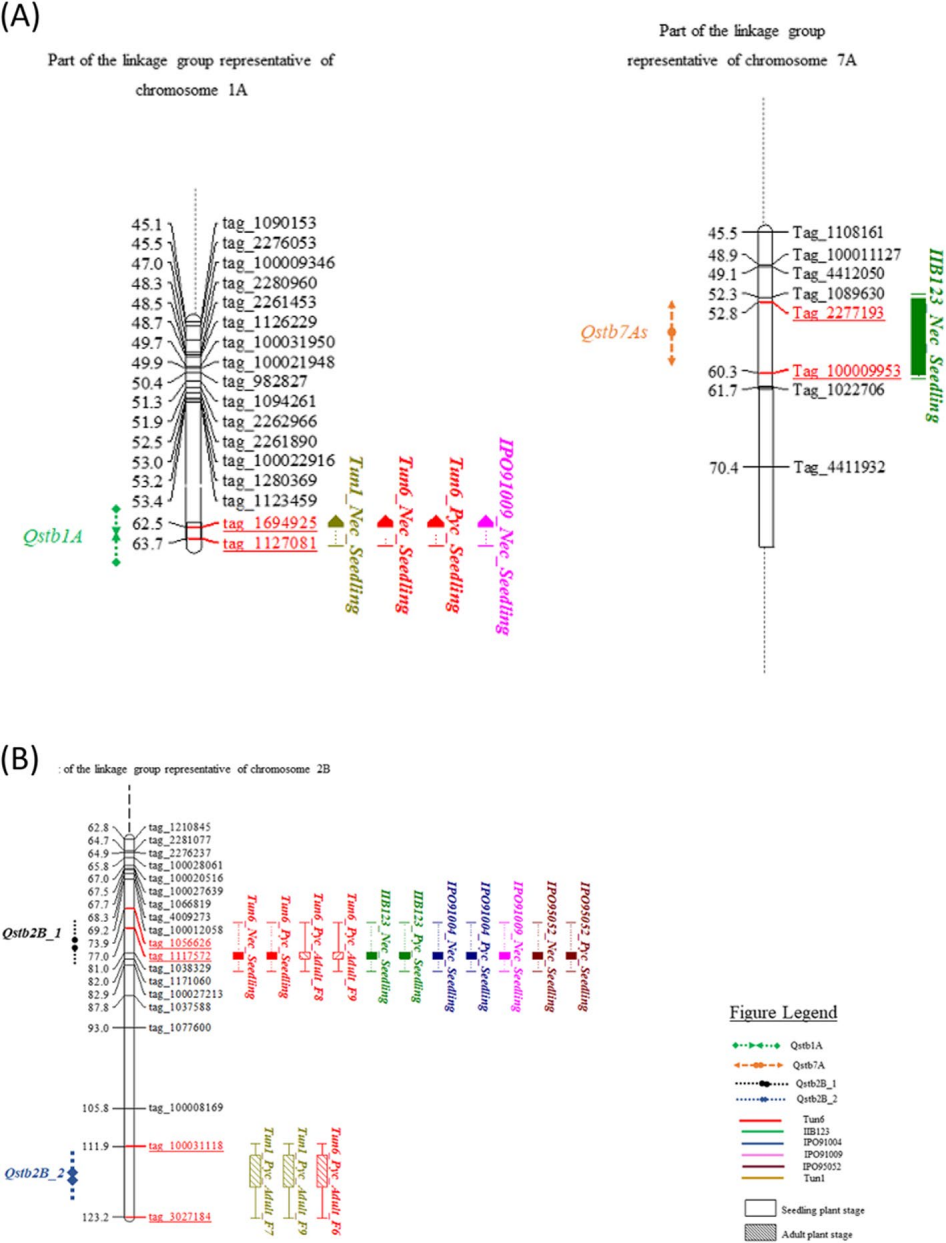
‘Agili39’/Khiar linkage groups associated with *Zymospetoria tritici* resistance. Panel a represents resistance QTL mapped at chromosomes 1A and 7A. Panel b represents the wide spectrum of resistance to *Zymoseptoria tritici* at the seedling and the adult plant stages detected in the ‘Agili39’/Khiar population on chromosome 2B. Marker intervals linked to these QTLs are underlined and written in red. The centiMorgan (cM) distances between marker loci and the marker loci IDs are on the left and right sides of the linkage map, respectively. Different colours are associated to *Zymoseptoria tritici* isolates. Empty and dashed square boxes refer to seedling and adult plant stages, respectively. Trait names are marked on top of each box

For the QTL analysis, the permutation test used to define the significant threshold LOD resulted into a LOD value of 3.5, hence only QTL with a LOD ≥ 3.5 were considered, which excluded all detected QTL with isolate IPO92003. In total, we identified four significant QTL on three chromosomes 1A, 7A and 2B. None of these QTL was mapped with every tested *Z. tritici* isolate, which underscores specificity of the interaction between *Z. tritici* and durum wheat. Two QTL were identified on chromosome 2B (Table 6; Fig. 3) designated as *Qstb2B_1* and *Qstb2B_2*. *Qstb2B_1* was effective in both the seedling stage—particularly against isolates Tun6, IIB123, IPO91009, IPO95052 and IPO91004, but not for isolates Tun1, IPO91018 and IPO92003—and the adult plant stage, where it provided resistance to *Z. tritici* isolate Tun6, but not to Tun1 and IIB-123 isolates. *Qstb2B_1* was mapped between the two *SNP* markers *Tag_1056626* and *Tag_111757* (Additional Table 4) with a confidence interval ranging between 69.5 and 75.5 cM and explained up to 61.6% of the phenotypic variance at the adult plant stage for Tun6 isolate, and up to 38.0% for IPO91004 isolate at the seedling stage (Table 6). LOD values of the *Qstb2B_1* QTL were variable depending on the isolate (Table 6). The highest LOD for the *Qstb2B_1* was registered for Tun6 at the F9 generation (33.9) at the adult plant stage, and for IPO91004 isolate for the necrosis score (17.3) at the seedling stage (Table 6).

**Table 6:** Detected quantitative trait loci (QTL) for *Zymoseptoria tritici* necrosis and pycnidia resistance in the seedling and the adult plant stages in the ‘Agili39’/Khiar recombinant inbred lines

Chromosome	QTL ID	Isolate	Trait – Physiological stage	LOD	Peak position (cM)	Flanking markers	Confidence Interval (cM)	PVE^a^ (%)	Additif effect
1A	*Qstb1A*	Tun1	Necrosis-Seedling	4.6	63	Tag_1694925—Tag_1127081	62.5—63	16.0	36.6
		Tun6	Necrosis-Seedling	4.1	63	Tag_1694925—Tag_1127081	62.5—63	9.1	50.9
			Pycnidia-Seedling	4.4	63	Tag_1694925—Tag_1127081	62.5—63	9.9	54.1
		IPO91009	Necrosis-Seedling	4.1	63	Tag_1694925—Tag_1127081	62.5—63	9.8	38.0
7As	*Qstb7A*	IIB123	Necrosis-Seedling	4.0	53	Tag_2277193—Tag_100009953	52.5—60.5	9.0	46.2
2B	*Qstb2B_1*	Tun6	Necrosis-Seedling	11.6	74	Tag_1056626 -Tag_111757	72.5—75.5	29.3	91.4
			Pycnidia-Seedling	11.9	74	Tag_1056626—Tag_1117572	72.5—76.5	29.6	93.7
			Pycnidia-Adult (F7:8)	23.4	75	Tag_1056626—Tag_1117572	74.5—76.5	52.9	1.5
			Pycnidia—Adult (F8:9)	33.9	74	Tag_105662—Tag_1117572	72.5—75.5	61.6	24.4
		IIB123	Necrosis-Seedling	12.9	74	Tag_1056626 -Tag_1117572	72.5—76.5	33.7	87.6
			Pycnidia-Seedling	5.4	74	Tag_1056626—Tag_1117572	70.5—76.5	17.5	38.2
		IPO91004	Necrsosis-Seedling	17.3	75	Tag_1056626—Tag_1117572	72.5—76.5	38.0	96.9
			Pycnidia-Seedling	7.2	74	Tag_1056626—Tag_1117572	69.5—76.5	21.2	52.5
		IPO91009	Necrosis-Seedling	9.5	74	Tag_1056626—Tag_1117572	73.5—76.5	24.6	60.1
		IPO95052	Necrsosis-Seedling	7.2	75	Tag_1056626—Tag_1117572	71.5—76.5	21.1	77.2
			Pycnidia-Seedling	3.2	74	Tag_1056626—Tag_1117572	72.5—76.5	10.0	36.0
	*Qstb2B_2*	Tun1	Pycnidia-Adult (F6:7)	4.8	117	Tag_100031118—Tag_3027184	107.5—123	14.9	0.6
			Pycnidia-Adult (F8:9)	5.5	113	Tag_100031118—Tag_3027184	106.5—118.5	17.1	9.1
		Tun6	Pycnidia-Adult (F5:6)	32.8	116	Tag_100031118—Tag_3027184	114.5—118.5	54.3	1.9

^a^Phenotypic Variance Explained

A second QTL, designated as *Qstb2B_2,* was mapped on chromosome 2B. This QTL was solely mapped at the adult plant stage for isolates Tun1 and Tun6, but not for the IIB123 isolate. This QTL is flanked by the two *SNP* markers *Tag_100031118* and *Tag_3027184* (Additional Table 4) at a confidence interval of 106.5 and 123 cM (Table 6). The highest LOD and explained variance were registered for the Tun6 isolate at the F9 generation with 32.8 and 54.3%, respectively (Table 6).Comparing the physical positions of the flanking markers linked to the *Qstb2B_1 and Qstb2B_2* QTL indicates that these two QTL are 5 Mb apart (Additional Table 4).

Two additional QTL with lower LODs and explained variances were mapped on chromosomes 1A and 7As designated as *Qstb1A* and *Qstb7A*, respectively and showed specificity for Tun1, Tun6 and IPO91009 isolates for *Qstb1A,* and for IIB-123 isolate for *Qstb7A* (Table 6, Fig. 3). These QTL were uniquely detected at the seedling plant stage. *Qstb1A* was flanked by the two *SNP* markers *Tag_1694925* and *Tag_1127081* (62.5 cM—63 cM), and explained up to 16.0% of the phenotypic variance with a maximum LOD of 4.6 for Tun1 isolate (Table 6, Additional Table 4). *Qstb7A,* mapped on chromosome 7A at 53 cM, was solely detected with IIB-123 isolate at the seedling plant stage for necrosis scores (Table 6). This QTL is flanked by the *SNP* markers *Tag_2277193* and *Tag_100009953* (Additional Table 4), with a LOD of 4.0 and explained 9% of the phenotypic variance (Table 6).

Overall, *Qstb2B_1* and *Qstb2B_2* had the widest efficacy compared to *Qstb1A* and *Qstb7A* (Fig. 3, Table 6). We also noticed that only *Qstb1A* and *Qstb2B_1* QTL were effective to both necrosis and pycnidia, whereas *Qstb7A* was only effective to necrosis (Table 6). All four QTL detected for resistance to *Z. tritici* in the ‘Agili39’/Khiar population were derived from the ‘Agili39’ parent (Table 6).

### Identification of epistatic QTL

The QTL interaction analysis has disclosed an epistatic QTL mapped on chromosome 5B, designated as *Qstb5B* (Table 7). Epistatic interactions were observed between the *Qstb5B* QTL and QTL *Qstb1A*, *Qstb7A*, *Qstb2B_1* and *Qstb2B_2*. *Qstb5B* was flanked between the two *SNP* markers *Tag_1125523* and *Tag_4909926* and was mapped at variable genetic position depending on the pairwise interaction ranging between 45 and 65 cM (Table 7)*.* Interestingly, the epistatic interactions were revealed with several isolates and at both stages (Table 7). Epistatic LODs and explained variances were variable and ranged between 6.8—29.1, and 0.9%—54.1%, respectively. The highest pairwise LOD was detected between *Qstb2B_2* and *Qstb5B* for the IPO95052 seedling pycnidia AUDPC score (LOD = 29). However, the Tun6 adult pycnidia score at F6 generation revealed the minimum epistatic LOD (6.8) among all detected pairwise interactions. Moreover, pairwise QTL interactions were mostly revealed by pycnidia AUDPC scores, except for the *Qstb2B_1*/*Qstb5B* interaction that was detected for necrosis and pycnidia AUDPC scores generated on the inoculated RILs by the Tun6 isolate at the seedling stage (Table 7).

**Table 7:** Epistatic analysis between quantitative traits loci in the Agili39/Khiar bi-parental mapping population using the inclusive composite interval mapping of digenic epistatic QTL (ICIM-EPI) method in QTL the IciMapping software

Isolate-Trait-Physiological Stage	Chromosome1 ^a^	QTL ID	Position 1 (cM) ^b^	Flanking markers (Position 1)	Chromosome 2 ^c^	QTL ID	Position 2 (cM) ^d^	Flanking markers (Position 2)	LOD^e^	PVE (%)^f^	Add1 ^g^	Add2 ^h^	AddbyAdd^i^
IPO95052-Pycnidia-Seedling	1A	Qstb1A	60	Tag_1123459—Tag_1694925	5B	Qstb5B	55	Tag_1125523—Tag_4909926	19.1	0.9	74.7	70.5	76.6
IIB123-Pycnidia-Seedling	7As	Qstb7A	55	Tag_2277193Tag_100009953	5B	Qstb5B	55	Tag_1125523—Tag_4909926	12.9	2.0	56.4	49.8	62.9
IIB123-Pycnidia-Adult (F9:10)		Qstb7A	75	Tag_4411932—Tag_4005129		Qstb5B	65	Tag_1125523—Tag_4909926	9.0	1.9	-0.1	0.0	0.8
IPO91009-Pycnidia-Seedling		Qstb7A	55	Tag_2277193—Tag_100009953		Qstb5B	55	Tag_1125523—Tag_4909926	19.5	1.1	59.4	-59.0	-56.8
IPO95052-Pycnidia-Seedling		Qstb7A	80	Tag_4411932—Tag_4005129		Qstb5B	50	Tag_1125523—Tag_4909926	19.8	0.9	74.3	73.1	75.0
Tun6-Necrosis- Seedling	2B	Qstb2B_1	75	Tag_1056626—Tag_1117572	5B	Qstb5B	60	Tag_1125523—Tag_4909926	7.3	31.0	94.4	-63.2	-76.5
Tun6-Pycnidia-Seedling		Qstb2B_1	75	Tag_1056626—Tag_1117572		Qstb5B	55	Tag_1125523—Tag_4909926	15.8	10.6	104.2	-83.8	-69.0
IPO91004-Pycnidia-Seedling		Qstb2B_1	70	Tag_100012058—Tag_1056626		Qstb5B	45	Tag_1125523—Tag_4909926	17.1	2.9	66.8	58.4	60.2
Tun6-Pycnidia-Adult (F5:6)		Qstb2B_2	115	Tag_100031118—Tag_3027184		Qstb5B	55	Tag_1125523—Tag_4909926	6.8	54.6	1.9	-0.4	-0.4
IIB123-Pycnidia-Seedling		Qstb2B_2	115	Tag_100031118—Tag_3027184		Qstb5B	55	Tag_1125523—Tag_4909926	15.2	1.8	26.8	54.4	57.3
IPO91009-Pycnidia-Seedling		Qstb2B_2	120	Tag_100031118 -Tag_3027184		Qstb5B	55	Tag_1125523—Tag_4909926	23.0	1.1	56.5	-57.7	-60.0
IPO95052-Pycnidia-Seedling		Qstb2B_2	100	Tag_1077600—Tag_100008169		Qstb5B	55	Tag_1125523—Tag_4909926	29.1	1.0	49.6	78.7	79.7

^a^Chromosome at the first scanning position

^b^Scanning position in cM of the first flanking marker pair

^c^Chromosome at the second scanning position

^d^Scanning position in cM of the second flanking marker pair

^e^LOD score caused by epistatic effects

^f^Phenotypic variation explained by epistatic effects

^g^Estimated additive effect of position 1

^h^Estimated additive effect of position 2

^i^Additive by additive epistatic effect at the two scanning positions. A positive value indicates that the effect of the parents’ effect is larger than the recombinant effect, and a negative value means that the recombinant effect is larger than the parents’ effect

**Fig. 4:**
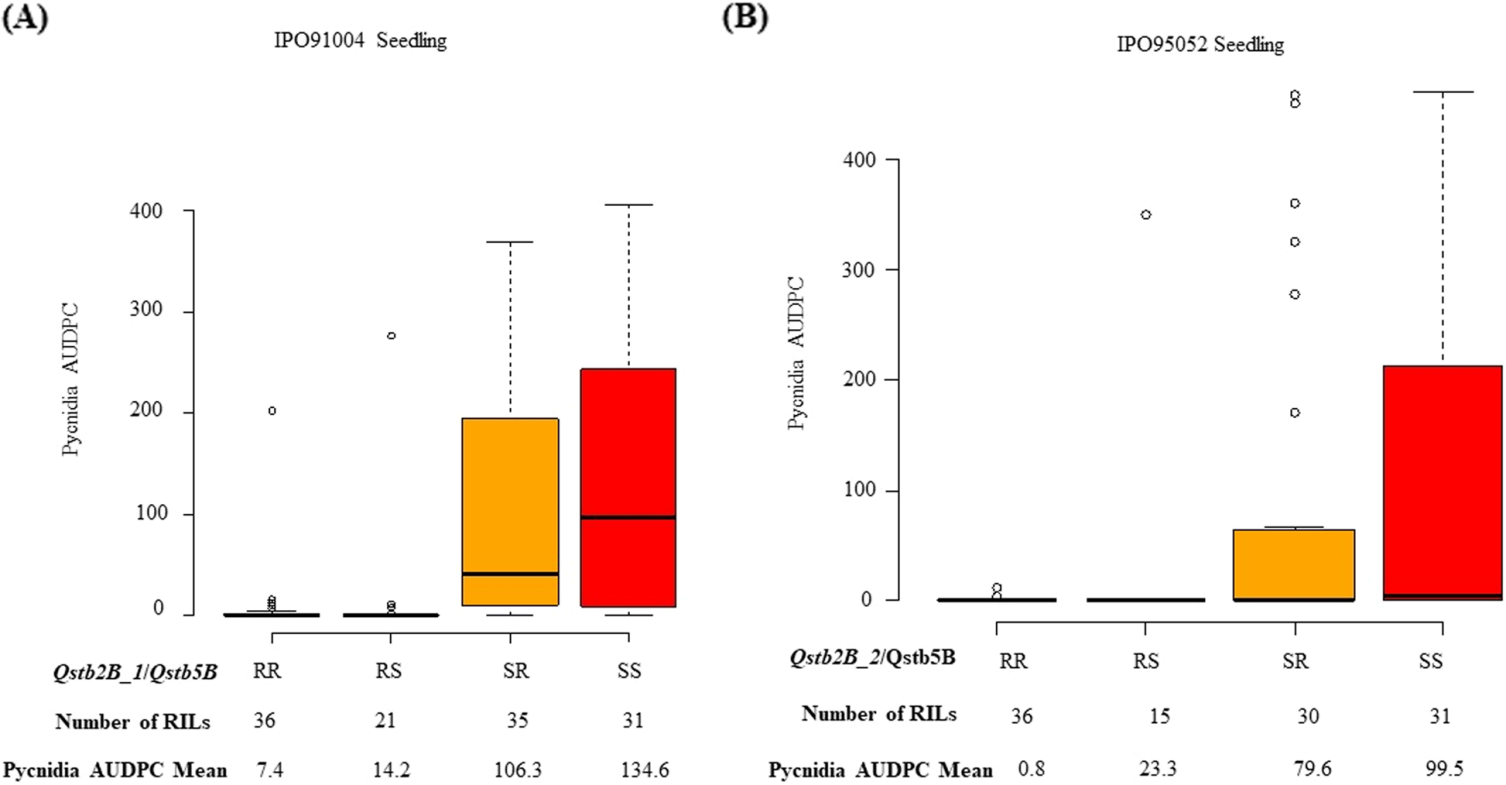
Epistatic interactions between QTL *Qstb2B_1* and *Qstb5B* (Panel A), and between *Qstb2B_2* and *Qstb5B* (Panel B). Box plots illustrate the significant effects of allele variants on pycnidia AUDPC on seedlings inoculated with the IPO91004 and the IPO95052 isolates. “R” and “S” denote the resistant and the susceptible alleles at each locus, respectively. The first allele refers to the *SNP* markers linked to *Qstb2B_1* or *Qstb2B_2*, however the second allele refers to the *Qstb5B SNP* marker. Number of genotypes and mean values of Pycnidia AUDPC are indicated under each allele classes. The black horizontal lines in the middle of the boxes are the median values for the Pycnidia AUDPC value in the respective allele classes. The vertical size of the boxes represents the inter-quantile range. The upper and lower whiskers represent the minimum and maximum values of data

Finally, we selected the pairwise epistasis *Qstb2B_1* / *Qstb5B* and *Qstb2B_2* / *Qstb5B* showing the highest epistatic LODs for a two-way interaction test to examine pycnidia AUDPC means of the variant allele combinations linked to QTL *Qstb2B_1*, *Qstb2B_2* and *Qstb5B* (Fig. 4). The first allele refers to the SNP markers linked to *Qstb2B_1* or *Qstb2B_2*, however the second allele refers to the *Qstb5B* SNP marker (Fig. 4).

Pycnidia AUDPC means were reduced when pairing the resistant alleles (RR) linked to *Qstb2B_1* /*Qstb5B QTL* and linked to *Qstb2B_2*/*Qstb5B* with pycnidia AUDPC scores of 7.4 and 0.8 for lines inoculated with the IPO91004 and IPO95052 *Z. tritici* isolates at the seedling stage, respectively (Fig. 4, panel A and B). Lines carrying the susceptible allele linked to the *Qstb5B* QTL (RS) showed an increase in pycnidia AUDPC scores compared to lines carrying both resistant allele (RR) (Fig. 4, panel A and B). Lines carrying solely the resistant allele linked to *Qstb5B* (SR) were rather susceptible showing high pycnidia AUDPC scores for both pairwise interactions Qstb2B_1/Qstb5B and *Qstb2B_2*/*Qstb5B*. This observation could be explained by the minor effect of the *Qstb5B* QTL in controlling pycnidia development, compared to the major effect of the *Qstb2B_1* and *Qstb2B_2* QTL in reducing pycnidia development.

## Discussion

*Zymoseptoria tritici* is a major threat to European and Mediterranean bread and durum wheat production [44]. Despite the increasing efforts to elucidate the genetic basis of tetraploid wheat resistance to STB [8, 40, 45], more studies are required for an effective breeding strategy in durum wheat for STB resistance.

In our study, all data indicated and confirmed significant ‘isolate’ and ‘line x isolate’ interactions as determined in earlier studies [37, 38, 46, 47]; and recently proven in the bread wheat – *Z. tritici* pathosystem where both *Stb6* and *AvrStb6* genes were cloned [48–50]. Moreover, and comparably to other cereal diseases, namely to rust [51–54], we determined QTL that are detected for either seedling (*Qstb1A* and *Qstb7A*) or adult plant stage (*Qstb2B_2*), as well as a QTL that was detected at both stages (*Qstb2B_1*). Our findings confirm that specific plant physiological stage resistances are commonly observed in the *Z. tritici* – wheat pathosystem [34]. Specific plant physiological stage resistances were also confirmed for other fungal diseases such as the powdery mildew and the leaf rust diseases [55, 56]. In fact, some *Z. tritici* resistance genes are uniquely effective at the seedling stage, such as the *Stb7* gene mapped in the spring wheat cultivar ST6 [57], or at the adult plant stage, such as the *Stb17* gene [58, 59]. In contrast, other resistance genes have proven to be effective at both seedling and adult plant stages alike the *Stb4* and *Stb5* qualitative genes [60, 61].

Subsequently, we compared the identified QTL to formerly identified *Z. tritici* genes using the reported literature. This comparison has revealed that a putative QTL for resistance to *Z. tritici* was mapped on chromosome 1A at 68 cM at the adult plant stage by Kidane et al. [8] through a genome-wide association study conducted on an Ethiopian durum wheat landrace population. Two other QTL mapped on chromosome 1A were also revealed by Goudemand et al. [62] in the bread wheat Apache/Balance population, and by Risser et al. [63] named as *QStb.lsa_fb-1A* in the bread wheat bi-parental mapping population Florett/Biscay. These QTL were mapped at the adult plant stage between 56 and 69 cM and could thus co-localize with the QTL mapped in the ‘Agili39’/Khiar population. However, and in contrast to the above-mentioned studies, the *Qstb1A* QTL mapped in the ‘Agili39’/Khiar population was solely detected at the seedling plant stage.

The *Qstb7A* QTL particularly conferred reduced necrosis values to *Z. tritici* isolate IIB123 and co-localizes with the major *Stb3* gene that was mapped in the bread wheat cultivar Israel 493 [64, 65].

Two QTL for STB resistance were mapped on chromosome 2B in the ‘Agili39’/khiar population. Other studies have also revealed genomic regions on chromosome 2B associated with the STB resistance [8, 62, 66–69].

The *Qstb2B_1* QTL identified in the ‘Agili39’/Khiar population likely co-localized with the known major gene *Stb9* that was mapped in the French bread wheat cv. Courtot [66]; with the *qSTB.2* QTL mapped in the Ethiopian durum wheat landrace population [8]; with the *QStb.ihar-2B.2* QTL mapped in the Liwilla/Begra bread wheat doubled-haploid population [68]; and with the *QStb.lfl-2B.1* mapped in the eight-founder MAGIC population of winter wheat [69]. However, the *Qstb2B_1* QTL is different from the QTL mapped at the long arm of chromosome 2B in the Nimbus/Stigg bread wheat mapping population [67].

The *Qstb2B_2* QTL likely co-localized with the QTL identified on chromosome 2B in the mapping populations Apache/Balance and FD3/Robigus [62] associated with both necrosis and pycnidia resistance in the adult plant stage. However, due to the unavailability of marker sequences, we cannot conclude that *Qstb2B_2* derived from ‘Agili39’ is the same locus that was mapped in the aforementioned bread wheat mapping populations. 

Thus, the identified QTL in ‘Agili39’ co-localized with previously mapped QTL for STB resistance in bread and durum wheat populations, hence we cannot claim a new *Stb* gene in the ‘Agili39’ landrace accession. However, we clearly have identified QTL conferring resistance to a wide range of *Z. tritici* isolates under artificial inoculation conditions in seedlings and adult plants, known as field resistance [70].

Thus far, in durum wheat, only partial resistance to *Z. tritici* was reported [16, 71]. Here, we derived *Qstb2B_1* from ‘Agili39’ that provides resistance to five *Z. tritici* isolates at the seedling stage, and to two isolates at the adult plant stage. *Qstb2B_1* explains up to 61.6% of the observed phenotypic variance and was characterized by a high heritability (0.98) with a dual action at the seedling and the adult plant stages. ‘Agili39’ is also the origin of *Qstb2B_2*, QTL providing a major adult resistance explaining up to 54.3% of the observed phenotypic variance. Our findings confirm that Tunisian durum landraces harbor highly effective *Z. tritici* resistance QTL.

In fact, the initial screening of the Tunisian landrace accessions showed a remarkable genetic diversity for STB resistance, as claimed also by Ouaja et al. [12] proving that Tunisian durum wheat landraces encompass diverse and valuable sources of resistance to *Z. tritici*. Eight landrace accessions (Agili37; Agili38; Agili39, Sbei99; Derbessi 12, Mahmoudi101, JK85 and Azizi27) were highly resistant and one landrace showed an intermediate response (‘Agili41’). The different ‘Agili’ landrace accessions reacted differently to the deployed *Z. tritici* isolates, suggesting a different genetic background, which is in accord with Ferjaoui et al. [72] study hypothesizing that the tested ‘Agili’ accessions most likely carry different *Stb* genes combinations. Hence, and alike other durum wheat landrace populations [8], the Tunisian durum wheat landraces uncover untapped allelic diversity that is of a great value to support effective breeding strategies to enhance STB resistance in durum wheat.

Our data demonstrated that the broad efficacy of the observed STB resistance in ‘Agili39’ is due to several stacked QTL, both at seedling as well as adult plant stages, which was also commonly observed in inheritance studies in bread wheat [34, 58]. Pyramiding genes for disease resistance has been an effective strategy in preventing boom-and-bust cycles, and is now amenable through marker assisted breeding as a strategy to maintain disease resistance durability, such as for wheat stem rust where various resistance gene combinations have well controlled the disease since the mid-1950s and more recently to the devastating Ug99 race [56, 73–75]. A concrete illustration for *Z. tritici* is the effective resistance to a wide range of isolates in the bread wheat germplasm ‘KK4500’ and ‘TE11’ which is conferred by stacking several known *Stb* genes [76–78] and also in other germplasm several QTL have contributed to broad efficacy of resistance [59]. Our data also confirm that stacking QTL in durum wheat results in broad efficacy of STB resistance. This study has identified genotypes harboring diverse resistance loci entailing dual actions at the different physiological stages constituting thus potential effective sources for *Z. tritici* resistance and will thus support sustainable breeding approach for *Z. tritici* resistance in durum wheat.

Finally, we explored QTL epistasis and identified four significant pairwise interactions of the identified QTL with an epistatic QTL mapped on chromosome 5B, designated as *Qstb5B*. Hence, the epistasis analysis has revealed other QTL that affects the expression of *Z. tritici* resistance in the ‘Agili39’/Khiar population. In fact, epistatic interactions between QTL are an important factor that affects the phenotypic expression of genes and genetic variation in populations [79–81]. Similarly, to many other studies [82–84], our data demonstrated interaction between QTL having main effect (*Qstb2B_1* and *Qstb2B_2*) that are involved in epistasis with the *Qstb5B* QTL affecting the same trait. The epistasis analysis showed an additive-by-additive effect between the *Qstb2B_1*/ *Qstb5B* QTL and the *Qstb2B_2*/*Qstb5B* QTL, with a major effect of QTL mapped on chromosome 2B (*Qstb2B_1* and *Qstb2B_2*) over the *Qstb5B* QTL. Interestingly, the epistasis analysis showed that the *Qstb2B_2* QTL, proven to control pycnidia development at the adult plant stage by QTL analysis, has also an effect in controlling pycnidia development at the seedling stage when interacting with the epistatic *Qstb5B* QTL. Nonetheless, the epistasis analysis did not indicate an interaction between the two major QTL *Qstb2B_1* and *Qstb2B_2* mapped on chromosome 2B.

## Conclusion

Our study deciphered ancient broad-based resistance to *Z. tritici* in a durum wheat landrace accession. A positive selection for the QTL linked markers may result in new high yielding durum wheat cultivars with wide resistance to *Z. tritici* reminiscent of the durable resistance to STB in landraces. Given the overall high susceptibility to STB in modern durum wheat cultivars, our data shed new light on disease resistance breeding in durum wheat.

## Methods

### Plant materials and study layout

Eleven durum wheat accessions (Table 8) and a bi-parental recombinant inbred (RIL) population derived from a single seed descent cross between the Tunisian landrace accession ‘Agili39’ and the high yielding commercial cv. Khiar, were screened for resistance to septoria tritici blotch. The RIL population was generated by crossing the resistant parent ‘Agili39’ to the susceptible Khiar following a single seed descent approach (SSD). The F1 plants were selfed to generate the F2 seeds. One head row of each F2 plants were then randomly selected and sown in one row to produce the F2 derived F3 plants (F3). This procedure was followed for all subsequent generations up to the F10 plants. Therecombinant population (Agili39’/Khiar) is maintained and available upon request at the National Institute of Agronomy -Tunisia (INAT) and at the CIMMYT gene banks.

**Table 8:** Nine Tunisian durum wheat landraces and two cultivars that were investigated for resistance to *Zymoseptoria tritici*

**Name**	Habitus	Source	Empiric evaluation of septoria tritici blotch under field conditions
Agili 37	landrace	INAT^b^	Resistant
Agili 38	landrace	INAT	Resistant
Agili 39 (P)^a^	landrace	INAT	Resistant
Agili 41	landrace	INAT	Resistant
Azizi 27	landrace	INAT	Resistant
Derbessi 12	landrace	INAT	Resistant
Jneh Khotifa 85	landrace	INAT	Resistant
Mahmoudi 101	landrace	INAT	Resistant
Sbei 99	landrace	INAT	Resistant
Khiar (P)^a^	cultivar	INRAT^c^	Susceptible
Karim	cultivar	INRAT	Susceptible

^a^Parents of the recombinant inbred population

^b^National Institute of Agronomy-Tunis, Tunisia

^c^National Institute of Agronomical Research-Tuni

For STB resistance screening, we performed three experiments (Table 2). The first experiment was repeated three times and comprised the screening of the 11 Tunisian landraces at the seedling stage (Z13.3/21) with a panel of 20 *Z. tritici* isolates (Table 2). The first experiment was performed to understand overall resistance patterns to STB and to select potential isolates for further screening of the RILs derived from the ‘Agili39’/Khiar cross, which consisted of the second experiment (experiment 2), performed thrice at the seedling stage. Finally, in the third experiment we tested the ‘Agili39’/Khiar population under field conditions in Oued-Bejá, located in North-Western Tunisia, over a period of five years, 2011 – 2014 and 2016 with three different *Z. tritici* isolates.

### Screening landraces and RILs population at the seedling stage for resistance to Zymoseptoria tritici

#### Experimental design, Plants management and growth conditions

A first experiment that consisted of the pre-screening of 11 Tunisian landrace accessions with 20 *Z. tritici* isolates was conducted at the seedling stage. The pre-screening assay followed a complete block design in three replicates and included the susceptible parent cv. Khiar, the susceptiblecv. Karim and the resistant parent ‘Agili39’ as checks (Table 2 and 8). This experiment enabled the selection of eight *Z. tritici* isolates that discriminated between the ‘Agili39’ and the cv. Khiar parents and were subsequently used to screen the ‘Agili39’/Khiar derived recombinant inbred lines (RILs) at the seedling stage.

For the seedling assay of the RIL population (experiment 2), we followed a split plot design with isolates as whole plots. Each whole plot consists of three neighbouring trays of fifty-four pots. The tested isolates were randomly allocated to the whole plots. Individual pots were the experimental units, and they were randomly arranged for each isolate/replicate combination on separate parallel greenhouse tables. ‘Replicate’, ‘isolate’, ‘line’ and the ‘isolate x line’ interaction were the fixed terms of our split plot model. However, the random term of the model consists of the ‘replicate x whole plot’ interaction. In all three replicates, eleven checks several checks were included with both parents ‘Agili39’ and cv. Khiar (Table 8).

Five seeds per pot per accessions were grown in VQB 7 × 7x8 cm plastic pots (TEKU®, Lohne, Germany), whereas 157 F6 RILs of the ‘Agili39’/Khiar mapping population were planted in round peat pots (Jiffy, Moerdijk, Netherlands), also five seeds per pot, using a special mixture for growing seeds (Substraat Zaai) provided by the greenhouse facility Unifarm of Wageningen University and Research (WUR), The Netherlands.

#### Zymoseptoria tritici isolates and inoculation procedures

Pre-cultures of each isolate were prepared in an autoclaved 100 ml Erlenmeyer flask containing 50 ml yeast glucose (YG) liquid medium (30 g glucose, 10 g yeast per litre distilled water). The flasks were inoculated with frozen isolate samples that were selected from different durum wheat growing countries and maintained at -80 °C in a *Z. tritici* isolate collection at WUR. The inoculated flasks were subsequently placed in an incubated rotary shaker (Innova 4430, New Brunswick Scientific, USA) set at 125 rpm and 15 °C for 5–7 days. These pre-cultures were then used to inoculate two 1L Erlenmeyer flasks containing 500 ml YG media per isolate that were incubated under the aforementioned conditions to provide sufficient inoculum for the seedling inoculation assays at growth stage (GS) 11 [85]. Spores were subsequently collected after overnight settling in static cultures, concentrated by decanting the supernatant medium, adjusted to 10^7^ spores ml-1 in a total volume of 40 ml for a set of 18 plastic pots or 24 Jiffy® pots and supplemented with two drops (µl/ml) of Tween 20 surfactant (MERCK®, Nottingham, UK).

Prior to the inoculation, plant development was allowed for 10 days in a greenhouse adjusted at a temperature of 18/16 °C (day/night rhythm) and relative humidity (RH) of 70%. Inoculations were conducted by spraying the inoculum over the seedlings that were placed in an inoculation cabinet on a rotary table, adjusted at 15 rpm, which is equipped with interchangeable atomizers and a water cleaning device to avoid cross- contamination. Infected plants were incubated in transparent plastic bags for 48 h under 100% RH. Post-inoculation conditions were set at a temperature of 22/ ± 2 °C and RH of ≥ 95%. Light intensity (son-T Agro 400 W lamps) and day length (16/8 h light/dark) were similar during pre- and post- inoculation conditions. Ten days after inoculation, seedlings were trimmed for the second and subsequent leaves to enable sufficient light on the inoculated primary leaves for appropriate disease development. Fertilizer (Sporumix PG®, Rotterdam, Netherlands; 0.5 g.l^1^) was applied to support plant growth.

#### Disease severity scoring in the seedling assay

In the seedling assays, disease severities were evaluated at 15, 18 and 21 days post-inoculation (dpi). These multiple observations enabled Area Under the Disease Progress Curve (AUDPC) calculations for quantitative analyses of temporal differences in disease progress. We estimated the quantitative presence of necrosis and pycnidia on the inoculated seedling leaves in percentages [36, 37, 86]. AUDPC calculations for seedling scores followed the trapezoidal method, which approximates the time variable and calculates the average disease intensity between each pair of adjacent time points [87].

### Screening RILs population at the adult plant stage for resistance to Zymoseptoria tritici

#### Experimental design and inoculation procedures

Adult plant assays of the ‘Agili39’/Khiar population were conducted at Oued-Bejá, located in North-Western Tunisia (36° 46′ 27.516'' N, 10° 3′ 36.432'' E), for five years, 2011 – 2014 and 2016, with Tun1 – Tun6 and IIB-123 *Z. tritici* isolates, respectively. This region belongs to the sub-humid bioclimatic zone of Tunisia with an average rainfall ranging from 500 to 850 mm and a daily mean temperature between 10–28 °C [72].

For the field trials, we adopted an augmented randomized complete block design. Five blocks with 1.5 m width and spaced 1.5 m were linearly drilled with 30 to 35 RILs, parents and four modern durum cultivars per block. Each line was sown as one spike per row of 1.5 m length and spaced 25 cm. We randomized all RILs, the parents and four additional checks modern durum wheat cvs. Karim, Nasr, Maali and Salim, important in Tunisian breeding programs and showing different levels of susceptibility in each block. For the 2016 field trial, we used a complete random block design with three replicates with both parents ‘Agili39’ and ‘Khiar’ as checks.

Field inoculations were conducted with three isolates (Tun1, Tun6 and IIB123) across the F5:6-F9:10 RILs generations. We used *Z. tritici* isolate Tun6 for three years to screen the F5:6 (*N* = 164), the F7:8 (*N* = 158) and the F8:9 (*N* = 157) RILs in 2011, 2013 and 2014, respectively and isolate Tun1 to screen the F6:7 (*N* = 158) in 2012 and the F8:9 (*N* = 157) in 2014. In 2016, we screened the F9:10 (*N* = 155) with *Z. tritici* isolate IIB123. In all field trials and across all generations, plants were inoculated twice. The first inoculation occurred at the tillering stage (GS 21–26) in order to initiate the disease infection, and the second inoculation was applied after 25–30 days when all RILs reached approximately the elongation stage (GS37) [85]. The second inoculation was applied to ensure an increase in the disease pressure. Field inoculations were conducted with a spore suspension adjusted to 10^7^ spores/ml of the corresponding *Z. tritici* isolate using a CO2-pressurized knapsack sprayer with a 1 m hand-held boom till run-off*.*

#### Disease severity scoring in the field tests

For the field evaluations, we scored pycnidial classes at 28 days post the second inoculation (GS 51) [85] that were later transformed to pycnidia percentages (0 = no pycnidia, 1 = 12%, 2 = 25%, 3 = 50%, 4 = 75% and 5 = 87%) [60, 88].

### Genotyping and construction of linkage map

Genomic DNA was extracted from fresh leaves using a modified CTAB (cetyltrimethylammonium bromide) method and quantified using NanoDrop 8000 spectrophotometer V 2.1.0. Whole-genome profiling was performed using DArT-Seq™ technology by Diversity Arrays Technology Pty Ltd, Australia, as described by Kilian et al. [89]. In brief, the DArT-Seq™ technology was optimized by selecting the most appropriate complexity reduction method for wheat (*Pst*I-*Mse*I restriction enzymes). DNA fragments digested with restriction enzymes were ligated with *Pst*I adaptors and unique barcodes, and then amplified following PCR. Amplicons were pooled and sequenced in a 96-multiplex on a HiSeq2000 (Illumina, USA) resulting in a total of 5,891 raw DArTSeq *SNP* markers.

The *SNP* markers were first filtered according to their polymorphism between the parents ‘Agili39’ and ‘Khiar’. A total of 3,459 filtered *SNPs* was subsequently tested for the segregation distortion that was determined by the calculation of the Chi-Square (*X* ^2^). Hence, *SNPs* with a major distortion of *p* < 0.001 were removed. Moreover, identical *SNPs,* which should be mapped to the same position on the linkage group, were identified (loci similarity = 1). Hence, only one marker of ‘similar loci’ was retained on the linkage map to reduce the calculation burden. The final set of filtered *SNPs* was then used to generate a genetic linkage map using JoinMap ® 4 software [90]. The regression mapping algorithm was used to construct the genetic map. Linkage between markers, recombination rate (Θ), and map distances was calculated using the Kosambi mapping function in JoinMap [91]. Markers were grouped using a minimum independence LOD (logarithm of the odds) score of 10.0 and linkage groups were established at a minimum LOD score of 3.0. Markers were linearly aligned in each linkage group, converting the recombination rates into the Kosambi's map distance in centimorgans. A sequential map builds up strategy was followed to determine the best marker position [92]. The best fitting position of markers was examined based on the goodness-of-fit test (chi-square) for the resulting map. The final map included bin markers that excluded similar *SNP* markers.

Linkage groups were subsequently assigned to chromosomes by aligning the *SNP* marker sequences of the linkage groups to the reference genome of the *Triticum turgidum subsp. durum Svevo.v1* and using the BLASTn function in the publicly available sequence database ‘Ensemble Plants’(https://plants.ensembl.org/Triticum_turgidum/Tools/Blast?db=core) [93]*.*

### Statistical analyses

#### Seedling data statistical analyses

Pycnidia AUDPC scores (P-AUDPC) of the pre-screening experiment (experiment 1) were analysed for their variance using the ‘*aov*’ function in the R environment [42] to test for the effect of ‘line’, the effect of ‘isolate’ and the effect of any ‘line x isolate’ interactions. The following linear model was fitted to the observed P-AUDPC scores:

$$Yijk= \mu +\alpha i+ \beta j+\left(\alpha \beta \right)ij+\epsilon ijk$$

where Yijk is the P-AUDPC score in the *Kth* replicate with isolate i and line j. μ is the overall mean. αi is the isolate i main effect. βj is the line j main effect. (αβ)ij is the interaction effect between isolate i and line j and ϵijk is the unexplained error.

For the split-plot seedling experiment performed on the RIL population (experiment 2), an analysis of variance for the necrosis and the pycnidia AUDPC scores (N-AUDPC and P-AUDPC) was performed using the *sp.plot* function available in the *Agricolae* package in the R environment [42, 94].Isolates were the whole-plot factors and RILs were the sub-plot factors. We fitted the observed N-AUDPC and P-AUDPC scores to the following linear model:

$$Y^{IJK}=\mu+\alpha i+\eta k\left(i\right)+\beta j+\left(\alpha\beta\right)ij+\epsilon ijk,$$

where *Y*_*ijk*_ is the N-AUDPC or P-AUDPC score in the *K*^*th*^ replicate of a plot with isolate i and RIL j. μ is the overall mean. αi is the fixed effect of isolate i. ηk(i) is the whole-plot error. βj is the fixed effect of RIL j. (αβ)ij is the interaction effect between isolate i and RIL j and ϵijk is the sub-plot error.

Significant differences between isolates were determined using the least significant difference (LSD) of N-AUDPC and P-AUDPC scores and using the *Agricolae* package in the R environment. ‘RIL x isolate’ grouping of necrosis and pycnidia AUDPC scores was defined based on the Bonferroni test at *p* < 0.05. Homogeneity of the seedling replicates was checked and homogeneous data across replications were subsequently averaged and used for the seedling QTL analysis [95].

Repeatability was estimated for the necrosis and pycnidia AUDPC generated at the seedling stage as follow:

$$Repeatability= \sigma 2G\left/ \left(\begin{array}{c}\sigma 2G+\frac{\sigma 2GE}{E}\\ +\frac{\sigma 2\varepsilon }{r}\end{array}\right)\right.$$

where σ^2^G is the variance component due to genotypes, σ^2^GE is the variance component due to the interaction between the genotype and the isolate, E is the number of isolates which is eight in the seedling experiment, σ^2^ε is the variance component due to the unexplained error and r is the number of replicates which is three per isolate for all seedling assays.

#### Field data statistical analyses

Field pycnidia scores generated by the inoculation of adult plants with Tun1 and Tun6 *Z. tritici* isolates were tested for their variance to check for the effect of ‘genotype’, ‘block’, ‘year’, ‘isolate’ and for any two-way and three-way interactions between the independent variable ‘genotype’ and the independent factors ‘block’, ‘year’ and ‘isolate’. A linear model was fitted to the observed pycnidia at the adult plant stage as follow:

$$Yijkt= \mu + \alpha i +\beta j+\gamma k+\eta t+\left(\alpha \beta \right)ij+\left(\gamma \beta \right)kj+\left(\eta \beta \right)tj+\left(\alpha \beta \eta \right)ijt+\varepsilon ijkt$$

where *Yijk*_*t*_ is the pycnidia coverage score of RIL j inoculated with isolate i in the *K*^*th*^ block at year t. μ is the overall mean. αi is the main effect of the isolate i. βj is the main effect of the RIL j. γk is the main effect of the block k. ηt is the main effect of the year t. (αβ)ij, (γβ)kj, (ηβ)tj are the two-way interaction effects of the RIL j with the isolate i, the RIL j with the block k and the RIL j with the year t, respectively. (αβη)ijt is the three-way interaction effect of RIL j with isolate i and with year t and finally, εijkt is the residual.

For the Tun1 and the Tun6 isolates tested on multiple consecutive years, a broad sense heritability (H^2^) was estimated as follow:

$$H2=\frac{\sigma 2G}{\sigma 2G+\frac{\sigma 2GE}{E}+\frac{\sigma 2\varepsilon }{r}}$$

where σ^2^G is the variance component due to genotypes. σ^2^GE is the variance component due to the interaction between the genotype and the isolate. E is the number of years which is 2 and 3 for Tun1 and Tun6 isolates, respectively. σ2ε is the variance component due to the unexplained error and r is the number of blocks which is five for all adult assays.

The least-square means (Lsmeans) of pycnidia coverages were derived from the individual year trials using the SAS software [96]. Transformed field data were subsequently considered for the field QTL analysis.

#### Correlation between seedling and adult plant assays and frequency distribution

A heatmap correlation matrix was calculated for the generated phenotypes at the seedling and the adult plant stages using the “Corr” function and visualized using the “corrplot” package in the R environment [43]. The “Corr” function was set to calculate the Pearson correlation. Positive and negative correlations were displayed in the heatmap matrix by a color gradient from red, indicating a positive correlation, to blue, indicating a negative correlation between the phenotypic traits. Frequency distribution figures were generated using the “hist” function in the R environment.

### QTL analysis procedure

An inclusive composite interval mapping of additive (ICIM-ADD) functionality in QTL IciMapping v4.1 [97] was used. Additive QTL were detected using a walk speed of 1.0 cM and the probability used in stepwise regression for additive QTLs was 0.001. The logarithm of odds (LOD) value of 3.0 was chosen to declare significant QTL, and the LOD value was calculated from 1000 permutations with type I error of 0.01. The phenotypic variance explained (PVE) and additive effect of individual QTL at the LOD peaks were also obtained. Identified QTL were plotted against their corresponding linkage groups using the MapChart © software version 2.3 [98]. Subsequently and for the identified QTL contributing to resistance, we aligned the linked *SNP* markers to the reference genome of the *Triticum turgidum subsp. durum Svevo.v1* and using the BLASTn function (https://plants.ensembl.org/Triticum_turgidum/Tools/Blast?db=core) in the publicly available sequence database ‘Ensemble Plants’ [93].

Epistatic interactions between QTL were identified by the inclusive composite interval mapping of digenic epistatic QTL (ICIM-EPI) method implemented in QTL IciMapping software v4.1 [97]. The LOD threshold was set at 5.00 to declare significant epistatic QTL and LOD value was calculated from 1000 permutations at the significance level of 0.05.

The highest epistasis interactions detected between the QTL (highest LOD) were subsequently selected for a two-way interaction test to examine pycnidia AUDPC means of the variant allele combinations linked to the epistatic QTL. Alleles linked to the detected QTL were named by ‘R’ and ‘S’ denoting the resistant and susceptible alleles, respectively. Hence, pycnidia AUDPC means of the four allele combinations ‘RR’, ‘RS’, ‘SR’ and ‘SS’ were examined and Boxplots were generated using the ‘*ggplot2*’ package in the R environment [99].

## Supplementary Information

**Additional file 1: Fig. S1.** Frequency distributions of the disease severity assessed as percentage pycnidia in seedlings of the F6 recombinant inbred lines of the ‘Agili39’/Khiar population. ‘A’ and ‘K’ are referring to the ‘Agili39’and cv. Khiar parents, respectively.

**Additional file 2: Table 1.** Analysis of variance of pycnidia percent of nine Tunisian durum landraces and two modern varieties inoculated with a diverse range of twenty durum derived *Zymoseptoria tritici *isolates. **Table 2.** Pearson correlation between the different tested isolates on the 'Agili39'/khiar population RILs at the seedling and the Adult plant stages. **Table 3.** Linkage groups, correspondent durum wheat chromosome, the average length indicated in centimorgans (cM), number of *SNPs* and the average inter-loci distance (cM) in the 'Agili39'/khiar genetic linkage map. **Table 4.** Sequences, genetic and physical positions of the flanking markers linked to the detected QTL on the 'Agili39'/Khiar mapping population.

## Data Availability

The used plant materials are available at the CIMMYT gene bank upon a reasonable request to the co-author Karim Ammar < k.ammar@cgiar.org > The sequencing raw data files generated during this study are uploaded to the CIMMYT data repository (https://hdl.handle.net/11529/10548618). The analyzed data are available as additional files to this article.
