# Supplementary material for: Deciphering resistance to Zymoseptoria tritici in the Tunisian durum wheat landrace accession ‘Agili39’
Source: BMC Genomics. 2022 May 17;23:372. doi: 10.1186/s12864-022-08560-2 (PMC9112612; doi:10.1186/s12864-022-08560-2)
Supplement: Supplementary file 1 — Additional file 1: Fig. S1. Frequency distributions of the disease severity assessed as percentage pycnidia in seedlings of the F6 recombinant inbred lines of the ‘Agili39’/Khiar population. ‘A’ and ‘K’ are referring to the ‘Agili39’and cv. Khiar parents, respectively. [file 12864_2022_8560_MOESM1_ESM.doc]

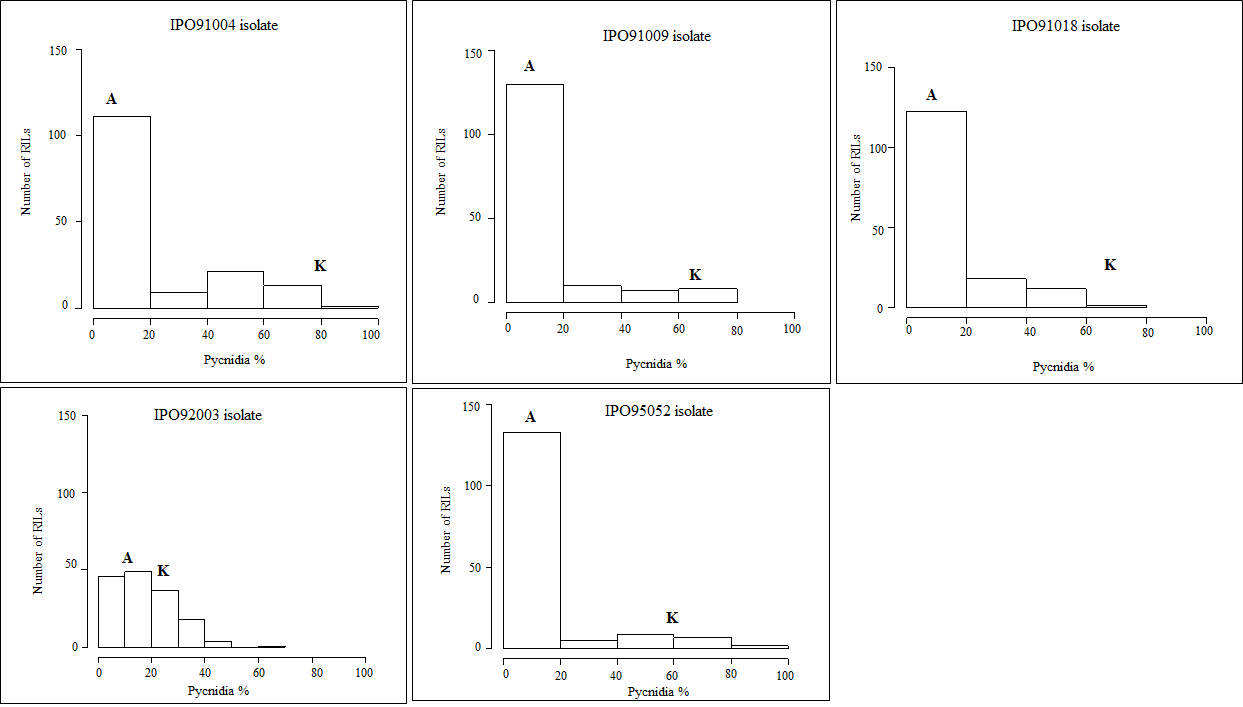


**Fig. S1** Frequency distributions of the disease severity assessed as percentage pycnidia in seedlings of the F6 recombinant inbred lines of the ‘Agili39’/Khiar population. ‘A’ and ‘K’ are referring to the ‘Agili39’and cv. Khiar parents, respectively
