# Supplementary material for: Deciphering resistance to Zymoseptoria tritici in the Tunisian durum wheat landrace accession ‘Agili39’
Source: BMC Genomics. 2022 May 17;23:372. doi: 10.1186/s12864-022-08560-2 (PMC9112612; doi:10.1186/s12864-022-08560-2)
Supplement: Supplementary file 2 — Additional file 2: Table 1. Analysis of variance of pycnidia percent of nine Tunisian durum landraces and two modern varieties inoculated with a diverse range of twenty durum derived Zymoseptoria tritici isolates. Table 2. Pearson correlation between the different tested isolates on the 'Agili39'/khiar population RILs at the seedling and the Adult plant stages. Table 3. Linkage groups, correspondent durum wheat chromosome, the average length indicated in centimorgans (cM), number of SNPs and the average inter-loci distance (cM) in the 'Agili39'/khiar genetic linkage map. Table 4. Sequences, genetic and physical positions of the flanking markers linked to the detected QTL on the 'Agili39'/Khiar mapping population. [file 12864_2022_8560_MOESM2_ESM.docx]

| **Additional Table 1**. Analysis of variance of pycnidia percent of nine Tunisian durum landraces and two modern varieties inoculated with a diverse range of twenty durum derived *Zymoseptoria tritici* isolates | | | | | | |
| --- | --- | --- | --- | --- | --- | --- |
| **Fixed terms** | **Df** | **Sum Sq** | **Mean Sq** | **F value** | **Pr(>F)** |  |
| **Isolate** | 19 | 54958 | 2893 | 97.16 | <2e-16 | *** |
| **accessions** | 10 | 69442 | 6944 | 233.26 | <2e-16 | *** |
| **Isolate x accessions** | 119 | 69149 | 581 | 19.52 | <2e-16 | *** |
| **Residuals** | 221 | 6579 | 30 |  |  |  |

| **Additional Table 2**. Pearson correlation between the different tested isolates on the 'Agili39'/khiar population RILs at the seedling and the Adult plant stages. | | | | | | | | | | | | | | | | | | | | | | | | |
| --- | --- | --- | --- | --- | --- | --- | --- | --- | --- | --- | --- | --- | --- | --- | --- | --- | --- | --- | --- | --- | --- | --- | --- | --- |
| Isolate | | IPO92003 | | IPO92003 | IPO95052 | IIB123 | IPO91004 | IPO91004 | IIB123 | IPO95052 | IPO91009 | Tun6 | Tun6 | IIB123 | Tun6 | Tun6 | Tun6 | Tun1 | Tun1 | IPO91018 | IPO91018 | IPO91009 | Tun1 | Tun1 |
| Trait | | N-S | P- S | | P-S | P-S | P-S | N-S | N-S | N-S | N-S | N-S | P-S | Field -F9 :10 | Field F8 :9 | Field – F5 :6 | Field- F7 :8 | Field-F6 :7 | Field-F8 :9 | N-S | P-S | P-S | N-S | P-S |
| IPO92003 | N-S | 1.0 |  | |  |  |  |  |  |  |  |  |  |  |  |  |  |  |  |  |  |  |  |  |
| IPO92003 | P-S | 0.6 | 1.0 | |  |  |  |  |  |  |  |  |  |  |  |  |  |  |  |  |  |  |  |  |
| IPO95052 | P-S | 0.0 | 0.0 | | 1.0 |  |  |  |  |  |  |  |  |  |  |  |  |  |  |  |  |  |  |  |
| IIB123 | P-S | 0.0 | 0.1 | | 0.8 | 1.0 |  |  |  |  |  |  |  |  |  |  |  |  |  |  |  |  |  |  |
| IPO91004 | P-S | -0.1 | 0.0 | | 0.7 | 0.9 | 1.0 |  |  |  |  |  |  |  |  |  |  |  |  |  |  |  |  |  |
| IPO91004 | N-S | -0.1 | 0.0 | | 0.4 | 0.6 | 0.7 | 1.0 |  |  |  |  |  |  |  |  |  |  |  |  |  |  |  |  |
| IIB123 | N-S | 0.1 | 0.1 | | 0.5 | 0.6 | 0.5 | 0.8 | 1.0 |  |  |  |  |  |  |  |  |  |  |  |  |  |  |  |
| IPO95052 | N-S | 0.1 | 0.1 | | 0.7 | 0.7 | 0.6 | 0.7 | 0.8 | 1.0 |  |  |  |  |  |  |  |  |  |  |  |  |  |  |
| IPO91009 | N-S | 0.1 | 0.1 | | 0.6 | 0.7 | 0.6 | 0.7 | 0.8 | 0.8 | 1.0 |  |  |  |  |  |  |  |  |  |  |  |  |  |
| Tun6 | N-S | 0.0 | 0.1 | | 0.6 | 0.6 | 0.5 | 0.7 | 0.8 | 0.8 | 0.8 | 1.0 |  |  |  |  |  |  |  |  |  |  |  |  |
| Tun6 | P-S | 0.0 | 0.1 | | 0.6 | 0.6 | 0.5 | 0.7 | 0.8 | 0.8 | 0.8 | 1.0 | 1.0 |  |  |  |  |  |  |  |  |  |  |  |
| IIB123 | Field F9 :10 | -0.1 | 0.0 | | 0.4 | 0.5 | 0.5 | 0.4 | 0.4 | 0.3 | 0.4 | 0.4 | 0.4 | 1.0 |  |  |  |  |  |  |  |  |  |  |
| Tun6 | Field F8 :9 | -0.1 | 0.1 | | 0.4 | 0.4 | 0.4 | 0.7 | 0.7 | 0.6 | 0.6 | 0.7 | 0.7 | 0.4 | 1.0 |  |  |  |  |  |  |  |  |  |
| Tun6 | Field-F5 :6 | -0.2 | 0.0 | | 0.3 | 0.4 | 0.4 | 0.5 | 0.5 | 0.4 | 0.5 | 0.6 | 0.5 | 0.4 | 0.8 | 1.0 |  |  |  |  |  |  |  |  |
| Tun6 | Field- F7 :8 | -0.2 | 0.0 | | 0.3 | 0.4 | 0.4 | 0.6 | 0.6 | 0.5 | 0.5 | 0.6 | 0.6 | 0.4 | 0.8 | 0.8 | 1.0 |  |  |  |  |  |  |  |
| Tun1 | Field-F6 :7 | 0.0 | 0.0 | | 0.4 | 0.5 | 0.5 | 0.4 | 0.3 | 0.3 | 0.4 | 0.4 | 0.3 | 0.4 | 0.4 | 0.4 | 0.4 | 1.0 |  |  |  |  |  |  |
| Tun1 | Field-F8 :9 | 0.0 | 0.0 | | 0.5 | 0.5 | 0.5 | 0.4 | 0.5 | 0.4 | 0.5 | 0.5 | 0.5 | 0.3 | 0.5 | 0.4 | 0.4 | 0.7 | 1.0 |  |  |  |  |  |
| IPO91018 | N-S | 0.1 | 0.0 | | 0.5 | 0.5 | 0.4 | 0.3 | 0.3 | 0.5 | 0.4 | 0.4 | 0.3 | 0.1 | 0.2 | 0.1 | 0.1 | 0.5 | 0.4 | 1.0 |  |  |  |  |
| IPO91018 | P-S | -0.1 | -0.1 | | 0.5 | 0.5 | 0.5 | 0.3 | 0.2 | 0.3 | 0.3 | 0.3 | 0.3 | 0.3 | 0.2 | 0.2 | 0.1 | 0.5 | 0.5 | 0.7 | 1.0 |  |  |  |
| IPO91009 | P-S | -0.1 | 0.0 | | 0.3 | 0.2 | 0.1 | 0.2 | 0.2 | 0.3 | 0.2 | 0.1 | 0.2 | 0.1 | 0.2 | 0.2 | 0.2 | 0.2 | 0.1 | 0.1 | 0.2 | 1.0 |  |  |
| Tun1 | N-S | 0.3 | 0.2 | | 0.2 | 0.3 | 0.1 | 0.2 | 0.4 | 0.4 | 0.5 | 0.4 | 0.4 | 0.0 | 0.2 | 0.0 | 0.1 | 0.2 | 0.3 | 0.5 | 0.4 | 0.2 | 1.0 |  |
| Tun1 | P-S | 0.1 | 0.1 | | 0.4 | 0.5 | 0.4 | 0.2 | 0.2 | 0.3 | 0.4 | 0.4 | 0.3 | 0.1 | 0.1 | 0.1 | 0.1 | 0.4 | 0.4 | 0.6 | 0.7 | 0.2 | 0.7 | 1.0 |
| _N- S = Necrosis at the seedling stage_ | | | | | | | | | | | | | | | | | | | | | | | | |
| _P-S = Pycnidia at the seedling stage_ | | | | | | | | | | | | | | | | | | | | | | | | |

| **Additional Table 3**. Linkage groups, correspondent durum wheat chromosome, the average length indicated in centimorgans (cM), number of *SNPs* and the average inter-loci distance (cM) in the 'Agili39'/khiar genetic linkage map. | | | | |
| --- | --- | --- | --- | --- |
| **Linkage group** | **Correspondant durum wheat chromosome** | **Total number of SNP markers** | **Lenght (cM)** | **cM/Locus** |
| 1 | 2B | 135 | 197.2 | 1.5 |
| 2 | 7B | 134 | 212.1 | 1.6 |
| 3 | 7B | 84 | 164.1 | 2 |
| 4 | 2B | 62 | 123.2 | 2 |
| 5 | 4A | 95 | 148.9 | 1.6 |
| 6 | 3B | 63 | 266.7 | 4.3 |
| 7 | 1B | 197 | 338 | 1.7 |
| 8 | 6A | 36 | 52.5 | 1.5 |
| 9 | 6B | 62 | 82.7 | 1.4 |
| 10 | 6A | 61 | 114.3 | 1.9 |
| 11 | 6B | 60 | 146.4 | 2.5 |
| 12 | 1B | 18 | 28.7 | 1.7 |
| 13 | 4B | 18 | 46 | 2.7 |
| 14 | 2A | 96 | 243.6 | 2.6 |
| 15 | 7A | 54 | 144.1 | 2.7 |
| 16 | 1A | 49 | 63.7 | 1.3 |
| 17 | 5A | 97 | 240.2 | 2.5 |
| 18 | 3A | 84 | 228.9 | 2.7 |
| 19 | 1B | 66 | 131.3 | 2 |
| 20 | 5B | 66 | 148.5 | 2.3 |
| 21 | 3A | 43 | 96.4 | 2.3 |
| 22 | 2A | 81 | 157 | 2 |
| 23 | 5B | 34 | 80.7 | 2.4 |
| 24 | 5B | 41 | 211 | 5.3 |
| 25 | 2B | 57 | 96.7 | 1.7 |
| 26 | 7A | 50 | 191.7 | 3.9 |
| 27 | 6A | 32 | 107.7 | 3.5 |
| 28 | 7A | 34 | 67.7 | 2.1 |
| 29 | 4B | 16 | 44.9 | 3 |
| 30 | 2A | 34 | 45.1 | 1.4 |
| **Total** | - | 1959 | 4220 | 2.3 |

| **Additional Table 4.** Sequences, genetic and physical positions of the flanking markers linked to the detected QTL on the 'Agili39'/Khiar mapping population | | | | | | | |
| --- | --- | --- | --- | --- | --- | --- | --- |
| **QTL ID** | **Flanking Markers** | **Genetic Position (cM)** | **Physical Position (Mb)^1^** | **Chromosome** | **SNP ^3^** | **SNP position** | **Sequence** |
| *Qstb1A* | Tag_1694925 | 62.5 | 489.685.941 - 489.685.993 | 1A | A/C | 49 | TGCAGCGCCAAATTGGAATGGCCATACCAATGACCCACCGAGAGGAAT[A/C]AAAAACAAATGTCTTACGAA |
|  | Tag_1127081 | 63.7 | NA^2^ |  | A/G | 8 | TGCAGGGG[A/G]AGGACGGCCAGGAGGAGGAGGACGACGAGGCAGAGGGAGGCGAGGAGAGGCCTCGGCCTG |
| Qstb7A | Tag_2277193 | 52.8 | 139.975.620 - 139.975.674 | 7A | A/G | 50 | TGCAGTGTGCCGACACGTCCTCGATCGAGACTTAGCTAAAAAAGCATATC[A/G]ACATCGTGAAGCGCAAGA |
|  | Tag_100009953 | 60.3 | 145.652.327 - 145.652.386 |  | T/A | 14 | TGCAGCATTACTG[A/T]TGATGATGATAACAGGAATCTACAAGTATATGTTTCATACATGATGAAGGGAGAT |
| *Qstb2B_1* | Tag_1056626 | 73.9 | 788.999.897 - 788.999.946 | 2B | G/C | 25 | TGCAGGTCAACAGCAATACATGGAC[C/G]ATCCTACGTAACCTGATGGCCCTGGAGGAGCAGATGACACGGC |
|  | Tag_111757 | 77 | 559.062.634-559.062.653 |  | C/T | 7 | TGCAGA[T/C]CCGACCCCCCGCACGACCACGACCACGACAACGCCCACAGGATCTCCTCGCCGCGCCGAGAT |
| Qstb2B_2 | Tag_100031118 | 111.912 | NA^2^ |  | C/T | 6 | TGCAGG[C/T]GCCGCAACCACAGCCGAGATCGGAAGAGCGGTTCAGCAGGAATGCCGAGACCGATCTCGTAT |
|  | Tag_3027184 | 123.209 | 783.725.692-783.725.730 |  | G/A | 8 | TGCAGCC[G/A]CATTGAGTCCTATGCACTTCGCGCACTACACGCCGAGATCGGAAGAGCGGTTCAGCAGGAA |
| ^1^ Physical positions were derived by blasting marker sequences against the *Triticum turgidum* reference genome cv. Svevo (RefSeq V 1.0) | | | | | | | |
| ^2^ No available hits on the reference genome Svevo (RefSeq V 1.0) on chromosomes 1A and 2B | | | | | | | |
| ^3^ The first allele corresponds to ‘Agili39’, and the second allele corresponds to cv. Khiar | | | | | | | |
